# Quaternized Carboxymethyl Chitosan-Based Silver Nanoparticles Hybrid: Microwave-Assisted Synthesis, Characterization and Antibacterial Activity

**DOI:** 10.3390/nano6060118

**Published:** 2016-06-17

**Authors:** Siqi Huang, Jing Wang, Yang Zhang, Zhiming Yu, Chusheng Qi

**Affiliations:** 1 Beijing Key Laboratory of Wood Science and Engineering, Beijing Forestry University, Beijing 100083, China; bjfu100514330@163.com (S.H.); bjfuwangjing@163.com (J.W.); qichusheng@bjfu.edu.cn (C.Q.); 2 MOE Key Laboratory of Wooden Material Science and Application, Beijing Forestry University, Beijing 100083, China

**Keywords:** quaternized carboxymethyl chitosan, uniform silver nanoparticles, microwave synthesis, thermal stability, antibacterial activity

## Abstract

A facile, efficient, and eco-friendly approach for the preparation of uniform silver nanoparticles (Ag NPs) was developed. The synthesis was conducted in an aqueous medium exposed to microwave irradiation for 8 min, using laboratory-prepared, water-soluble quaternized carboxymethyl chitosan (QCMC) as a chemical reducer and stabilizer and silver nitrate as the silver source. The structure of the prepared QCMC was characterized using Fourier transform infrared (FT-IR) and ^1^H nuclear magnetic resonance (NMR). The formation, size distribution, and dispersion of the Ag NPs in the QCMC matrix were determined using X-ray diffraction (XRD), X-ray photoelectron spectroscopy (XPS), ultraviolet-visible (UV-Vis), transmission electron microscopy (TEM), and field emission scanning electron microscope (FESEM) analysis, and the thermal stability and antibacterial properties of the synthesized QCMC-based Ag NPs composite (QCMC-Ag) were also explored. The results revealed that (1) QCMC was successfully prepared by grafting quaternary ammonium groups onto carboxymethyl chitosan (CMC) chains under microwave irradiation in water for 90 min and this substitution appeared to have occurred at -NH_2_ sites on C2 position of the pyranoid ring; (2) uniform and stable spherical Ag NPs could be synthesized when QCMC was used as the reducing and stabilizing agent; (3) Ag NPs were well dispersed in the QCMC matrix with a narrow size distribiution in the range of 17–31 nm without aggregation; and (4) due to the presence of Ag NPs, the thermal stability and antibacterial activity of QCMC-Ag were dramatically improved relative to QCMC.

## 1. Introduction

Silver nanoparticles (Ag NPs) have recently received extensive attention from researchers and developers due to their attractive optical, electronic, and catalytic properties and excellent antimicrobial activity [1,2,3]. Ag NPs exhibit strong antibacterial activity toward a broad range of microorganisms [4], but simultaneously possess a remarkably low human toxicity. These properties have caused these new materials to be recognized as one of the most powerful antimicrobial agents of the 21st century, resulting in their wide use in the biomedical field, food packaging, coating, clothing, textile, and other applications [5,6].

Numerous methods have been developed to prepare Ag NPs, including chemical reduction, thermal decomposition, UV irradiation reduction, photoreduction, electrolytic process, gamma irradiation, and others [7]. In these methods, the physical approaches require highly sophisticated instruments and specific conditions [8], while chemical reduction offers the superiority of simple processibility making it widely used. However, conventional chemical reduction methods require reducing agents including sodium borohydride (NaBH_4_), hydrazine hydrate (HHA), or other organic compounds in addition to stabilizing agents such as triphenylphosphine, polyvinylpyrrolidone, and sodium citrate. Many of these reagents have potential environmental toxicity and biological risks [9,10]. The increasing awareness of environmental protection has led to the development of an eco-friendly approach for the synthesis of Ag NPs using judicious choices of reducing and stabilizing agents and solvents.

From the perspective of green synthesis, the biopolymer chitosan has attracted extensive research interest for the preparation of Ag NPs. Chitosan is the second most abundant natural polysaccharide with β-1,4-linked N-acetyl-D-glucosamine and D-glucosamine units [11,12], which possess largely free amino and hydroxyl groups offering a template for growing nanometals. Wei *et al.* synthesized Ag NPs by reducing cationic silver using polycationic chitosan, but this process was time-consuming and the resulting Ag NPs varied in size [2]. In effort to prepare uniform Ag NPs rapidly, An *et al.* used carboxymethyl chitosan (CMC) as a stabilizer, but an additional reducing agent NaBH_4_ was also required [13].

Quaternized carboxymethyl chitosan (QCMC) is a novel amphoteric polymer synthesized by grafting carboxymethyl groups and quaternary ammonium groups onto chitosan chains [14]. Favored for its properties of good water solubility, antimicrobial effect, biodegradability, biocompatibility, high moisture-absorption and moisture-retention, QCMC is widely applied in antioxidant, drug encapsulation, tissue engineering, as well as the cosmetics and antibacterial fields [15,16]. What’s more, QCMC is considered a promising green reducing and stabilizing agent for the preparation of nanometals. It has been deduced that the large number of hydroxyl groups, carboxyl groups and quaternary ammonium groups on the chains of QCMC makes it remarkable as a reducing agent, and it also shows great stabilizing performance due to the surfactant-like structure [17,18].

The use of microwave heating for chemical synthesis, pioneered by Gedye, is faster and simpler than similar thermal transfer methods and it is especially appropriate for nanometal synthesis, because it provides uniform heating around the nanoparticles and can assist in their ripening without aggregation [19,20]. Therefore, the combination of microwave heating with a green reducing and stabilizing agent can open a new pathway for the synthesis of Ag NPs.

In the present study, a rapid, simple and totally green method was developed to synthesize uniform, stable Ag NPs using laboratory-prepared quaternized carboxymethyl chitosan (QCMC) as a reducing and stabilizing agent. The structure of the prepared QCMC was characterized and the formation, size distribution, and dispersion of the Ag NPs in the QCMC matrix were investigated in detail.

From another point of view, the presence of Ag NPs may also enhance the thermal stability and antimicrobial activity of the QCMC, resulting in outstanding hybrid performance. However, to the best of our knowledge, there have been relatively few reports in the literature of the properties of QCMC-based Ag NPs composite (QCMC-Ag). Thus, the thermal stability and antibacterial capability of the synthesized QCMC-Ag were also explored.

## 2. Materials and Methods

### 2.1. Materials

**Figure 1:**
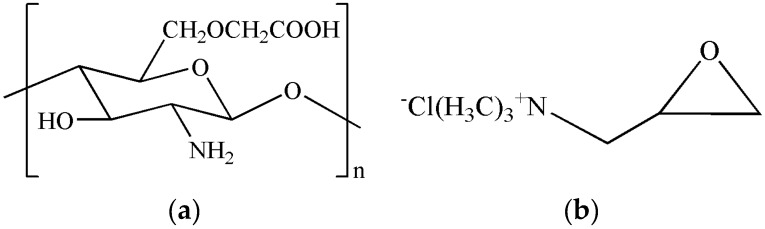
Chemical structure of (**a**) carboxymethyl chitosan (CMC) and (**b**) 2,3-epoxypropyltrimethyl ammonium chloride (ETA).

CMC was obtained from Zhejiang Biochemical Co. (Taizhou, China) and was used as received. The molecular weight of the CMC was 100 kDa, the degree of substitution of the carboxymethyl groups was 0.91, and the degree of deacetylation was 90.2%. The 2,3-epoxypropyltrimethyl ammonium chloride (ETA) was provided by Shanghai Dibo Chemical Technology Co., Ltd. (Shanghai, China). Silver nitrate was purchased from Guangdong Guanghua Science and Technology Co., Ltd. (Guangdong, China). An XH-100A microwave synthesis system was obtained from Beijing Xiang-Hu Technology Co., Ltd. (Beijing, China). Figure 1 shows the chemical structure of CMC [21,22] and ETA. All other reagents and solvents were of analytical grade and were used as received.

### 2.2. Preparation of QCMC

The QCMC was prepared based on a reference with modification [16]. The synthesis was conducted in the microwave unit using ETA as the quaternized modification agent. Five grams of CMC powder were dissolved in distilled water with ultrasonic stirring for 30 min and then placed in the microwave synthesis equipment. The ETA was dissolved in 50 mL of distilled water and dropped slowly into the CMC solution under microwave irradiation for 1000 W and reacted at 80 °C for 90 min. The mass ratio of the ETA to CMC was six to one. Next, the reaction mixture was precipitated and washed three times using pure ethanol. After dialyzing the mixture against deionized water for 7 days, the resultant product was freeze-dried at −60 °C and ground to a powder in a glass mortar. The molecular weight of QCMC determined using GPC was 98 kDa and the degree of substitution of the quaternary ammonium groups was 0.89 which was determined by potentiometry [23].

### 2.3. Preparation of the QCMC-Based Ag NPs Composite

The QCMC powder was dissolved in distilled water to prepare a 2 mg/mL solution after ultrasonication for 10 min. Silver nitrate was dissolved in 10 mL of distilled water to obtain a 0.1 mmol/mL solution, and then was added drop-wise to the QCMC aqueous solution with magnetic stirring at room temperature. Next, the mixture was heated via microwave irradiation at 1000 W at 70 °C for 8 min. A QCMC-based Ag NPs composite was obtained by dialysis against distilled water until silver ions could not be detected in the dialysate using 0.1mol/L HCl solution. The product was then lyophilized at −60 °C. The ratio of the QCMC to AgNO_3_ was 500 mg: 1 mmol. The final composite was designated as QCMC-Ag.

### 2.4. Characterization

Waters-1515 gel permeation chromatograph (GPC, Waters, MI, USA) was used to evaluate the molecular weight of the products. Fourier transform infrared (FT-IR) spectra were obtained using a Vertex 70v FT-IR spectrophotometer (Bruker, Karlsruhe, Germany). ^1^H NMR spectra were recorded on an ASCEND 400 spectrometer (Bruker, Karlsruhe, Germany). X-ray diffraction (XRD) patterns were measured using a D8 advance X-ray diffractometer (Bruker, Karlsruhe, Germany); the relative intensity was recorded in the scattering range (2θ) of 5°–90°. X-ray photoelectron spectroscopy (XPS) spectra were obtained using PHI 5000CESCA System surface analysis equipment (PHI Co., Chanhassen, MN, USA) with Mg-K_α_ X-ray source (hγ = 1253.6 eV). Ultraviolet-Visible (UV-Vis) spectra were recorded in the range of 300–600 nm using UV2310II UV-Vis spectrophotometer (INESA, Shanghai, China). JEOL-2100F transmission electron microscopy (TEM, JEOL, Tokyo, Japan) was used to investigate the microstructure and size distribution of the Ag NPs. The size distribution was statistically analyzed for 300 randomly selected nanoparticles using the public software Image J. Surface morphology was observed via an SU8010 field emission scanning electron microscope (FESEM, Hitachi, Japan).

### 2.5. Thermal Stability Experiment

Thermal stability was evaluated by thermogravimetric analysis (TGA) using a TGAQ50 thermal analyzer (TA, New Castle, DE, USA). TGA and derivative thermogravimetry (DTG) thermograms were obtained in the temperature range of 30 to 700 °C under nitrogen and with a ramp rate of 10 °C/min.

### 2.6. Antibacterial Assay

To study the antibacterial property of the QCMC-based Ag NPs composite, gram-positive *Staphylococcus aureus* (provided by the Chinese Academy of Sciences) served as model pathogenic bacteria. The antibacterial test was performed according to our previous report [24]. Antibacterial solutions were diluted to appropriate concentrations in phosphate buffer (pH 7.2), and 10 mL of each sample was added to sterile petri dishes containing 10 mL of nutrient agar solution. *Staphylococcus aureus* was adjusted by sterile distilled water to 10^7^ cfu/mL. A bacterial suspension of 2 μL was then inoculated on the prepared nutrient medium-containing antibacterial suspension and incubated at 37 °C. The minimum inhibition concentration (MIC) values were measured after a 24 h culture. 

The concentration of silver released from QCMC-Ag was analyzed using a Thermo Scientific iCAP 6500 Duo inductively coupled plasma-optical emission spectrometer (ICP-OES, TMO, Cambridge, UK). To measure the silver release, the QCMC-Ag (approximately 0.05 g) was immersed in 10 mL of phosphate buffer (pH 7.2) and kept at 37 °C (the same procedure carried out during antibacterial test). After 24 h of release, the above sample was centrifugated for 40 min at the speed of 4800 rpm, then 1 mL of the supernatant was taken out and diluted with concentrated solutions of HCl and HNO_3_. The diluted supernatant was used for inductively coupled plasma optical emission spectroscopy (ICP-OES) analysis.

## 3. Results and Discussion

### 3.1. Structure Characterization of QCMC

**Figure 2:**
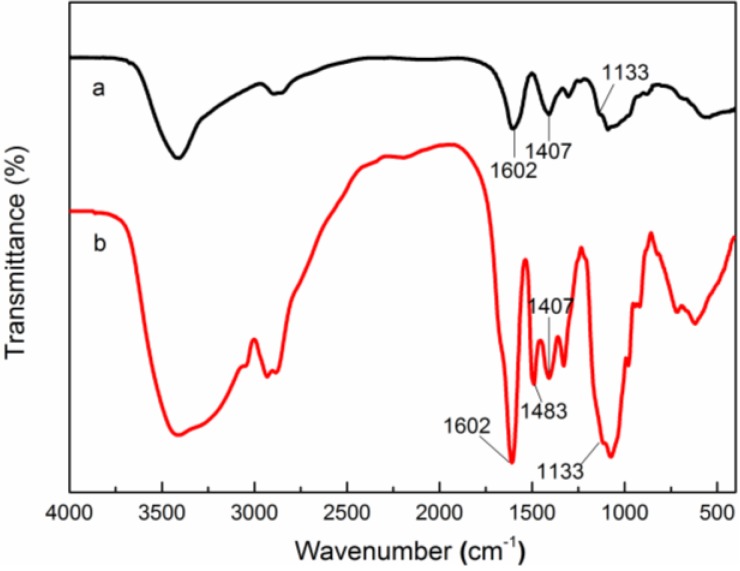
Fourier transform infrared (FT-IR) spectra of (**a**) carboxymethyl chitosan (CMC) and (**b**) Quaternized carboxymethyl chitosan (QCMC).

The FT-IR spectra of CMC and QCMC are shown in Figure 2. In the characteristic peaks of CMC (Figure 2a), the absorption peaks at 1602 and 1407 cm^−1^ correspond to the asymmetrical and symmetrical stretching vibrations of COO^−^ group. In the QCMC spectrum shown in Figure 2b, the characteristic peaks of CMC are present and a new adsorption band appeared at 1483 cm^−1^ that belonged to the methyl groups of the quaternary ammonium salt, confirming the existence of the C6-substituted carboxymethyl groups and quaternary ammonium groups on the QCMC chains. In addition, compared to CMC, the peak at around 1133 cm^−1^, which belonged to the C–O stretching band of the secondary hydroxyl group, is unchanged in QCMC, verifying that quaternization did not occur at the C3 position. These results imply that the quaternization of CMC and the substitution may occur at -NH_2_ sites on C2 position of the pyranoid ring, which is consistent with previous studies [25,26,27].

**Figure 3:**
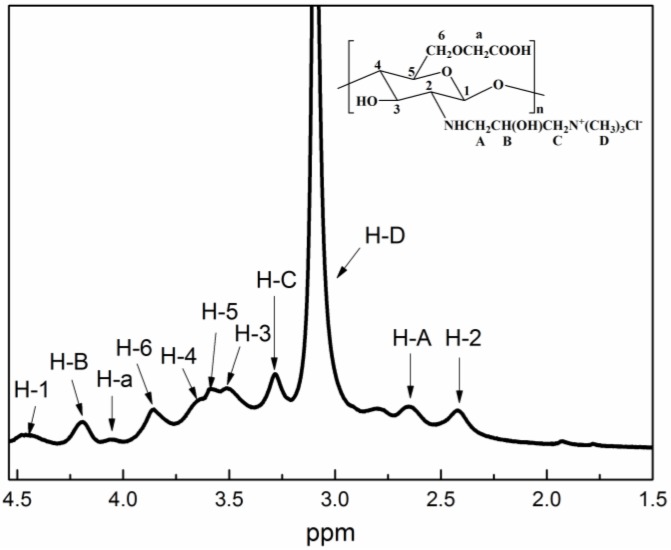
^1^H nuclear magnetic resonance (NMR) spectrum of QCMC.

**Figure 4:**
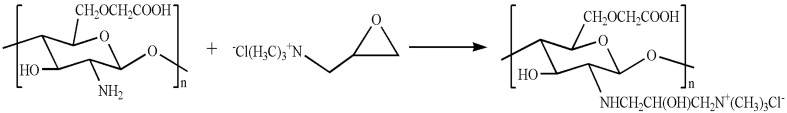
The reaction illustration of QCMC.

To verify the QCMC structure, the ^1^H NMR spectrum was investigated as shown in Figure 3. In the spectrum of QCMC, the peaks at δ = 4.44, 2.42, 3.51, 3.64, 3.58 and 3.85 ppm correspond to H–1, H–2, H–3, H–4, H–5 and H–6. The signal at δ = 4.06 ppm can be attributed to the protons of the C6-substituted carboxymethyl groups. The most intensive signal at δ = 3.10 ppm can be attributed to the methyl protons of the quaternary ammonium salt, which agrees with the report by Cai *et al*. [25]. The chemical shifts at 2.65, 4.19 and 3.28 ppm belong to H–A, H–B and H–C of the C2-substituted quaternary ammonium groups. The ^1^H NMR results are in agreement with previous studies [16,26], which further demonstrated that QCMC was successfully synthesized under microwave irradiation in water. The possible mechanism for the preparation of QCMC is illustrated in Figure 4.

### 3.2. Characterization of the QCMC-Based Ag NPs Composite

#### 3.2.1. XRD Analysis

**Figure 5:**
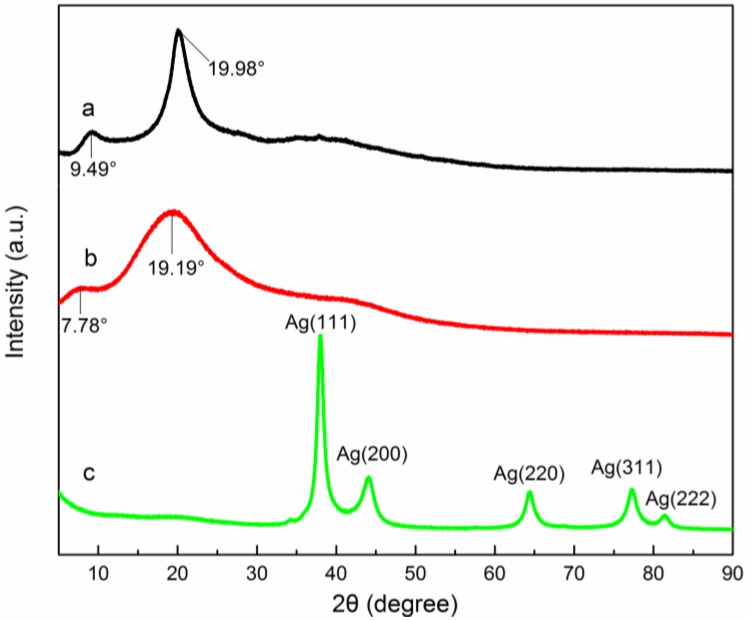
X-ray diffraction (XRD) patterns of (**a**) CMC, (**b**) QCMC and (**c**) QCMC-Ag.

The XRD method is widely used to examine the crystallographic nature of a material to confirm the formation of nanoparticles [28]. The XRD patterns of CMC, QCMC and QCMC-Ag are shown in Figure 5. In the XRD pattern of QCMC-Ag (Figure 5c), the diffraction peaks at 2θ = 38.01, 44.14, 64.47, 77.21, and 81.39 are due to reflections from (111), (200), (220), (331), (222) planes of metallic silver with a face centred-cubic lattice structure (JCPDS 04-0783), which indicate that pure well-crystallized silver was obtained via the proposed facile and green method. In the reported experiment, no other agents were added to the reaction system implying that QCMC solution played a vital role in the synthesis of the silver crystals. QCMC possesses many hydroxyl, carboxymethyl, and quaternary ammonium groups, which have a relatively good reducing capability [1,29].

In the XRD pattern of the CMC (Figure 5a), two major characteristic crystalline peaks were observed near 9.49° and 19.98°, which indicate crystallization. The crystalline peaks shifted towards a lower angle and became weaker in the QCMC, while they disappeared entirely in the XRD pattern of the QCMC-Ag. Therefore, we deduced that the crystal structure of the original polymer remained in the QCMC but was destroyed in the QCMC-Ag. This result can be explained by the embedding of the silver particles in the QCMC polymer network, which disrupted the regularity of the polymer chains and the intramolecular hydrogen bonding in polymer molecules, resulting in significant changes in the polymer’s crystalline structure.

#### 3.2.2. XPS Analysis

**Figure 6:**
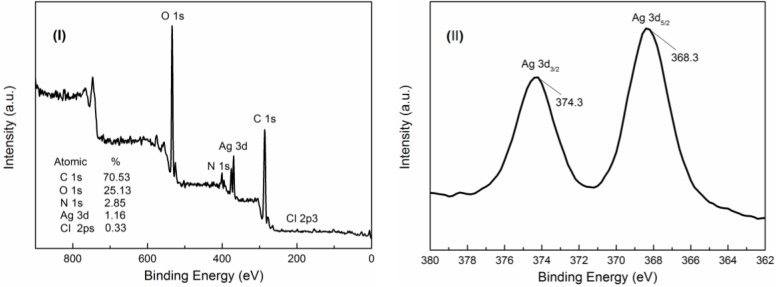
X-ray photoelectron spectroscopy (XPS) spectra of as-prepared QCMC-Ag. (**I**): Survey-scan spectrum. (**II**): High-resolution Ag 3d spectrum.

XPS analysis was conducted on the product to further determine the chemical composition and the valence states of the prepared material [28,30]. A survey-scan XPS spectrum of QCMC-Ag (Figure 6I) revealed that the sample was composed of 70.53% atomic weight carbon, 25.13% oxygen, 2.85% nitrogen, 1.16% silver, and 0.33% chlorine. The high-resolution XPS result for silver is shown in Figure 6II. The XPS signals for Ag 3d show two clear peaks attributed to Ag 3d_5/2_ (368.3 eV) orbit and Ag 3d_3/2_ (374.3 eV) orbit. Both of these peaks at 368.3 and 374.3 eV are associated with Ag^0^ [31,32,33,34], indicating that the sample was composed of a single phase of silver, which is in good accordance with the XRD results shown in Figure 5. Based on the results of the XPS analysis, it can be concluded that pure metallic silver was synthesized as a result of the reaction, further confirming the complete reduction of Ag^+^ to Ag^0^ using QCMC as the reducing agent.

#### 3.2.3. UV-Vis Spectroscopy Analysis

**Figure 7:**
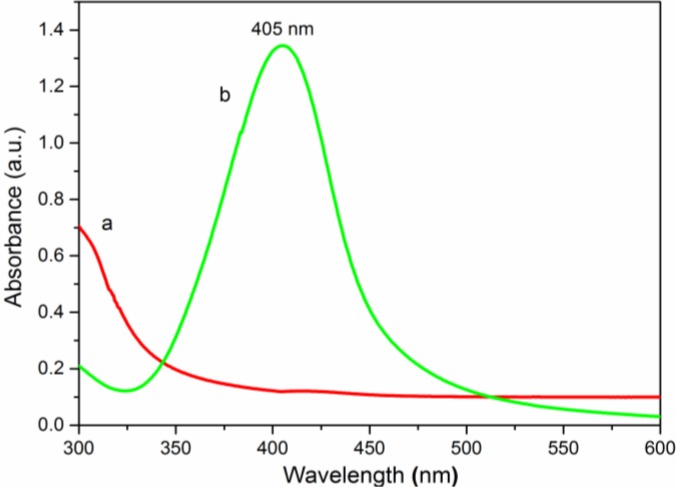
Ultraviolet-visible (UV-Vis) spectra of (**a**) QCMC and (**b**) QCMC-Ag.

UV-Vis spectroscopy is a simple and sensitive technique for the characterization of Ag NPs thanks to its singular excitation of surface plasmon resonance (SPR) in the UV-Visible region [18]. The formation and size distribution of Ag NPs can be easily revealed using a UV-Vis spectrophotometer [35]. As shown in Figure 7a, original QCMC exhibited no peaks in the range of 300–600 nm, but QCMC-Ag (Figure 7b) displayed the unique SPR absorption band for the Ag NPs at 405 nm, verifying that Ag^+^ was reduced to Ag^0^ and formed Ag NPs in the QCMC. In addition, the sharp SPR absorption peak indicates a very narrow distribution in the particle size [36,37]. The shape of the SPR absorption band was symmetrical and quite narrow, suggesting that the Ag NPs were spherical and monodispersed [38].

#### 3.2.4. Morphology Analysis (TEM and FESEM)

**Figure 8:**
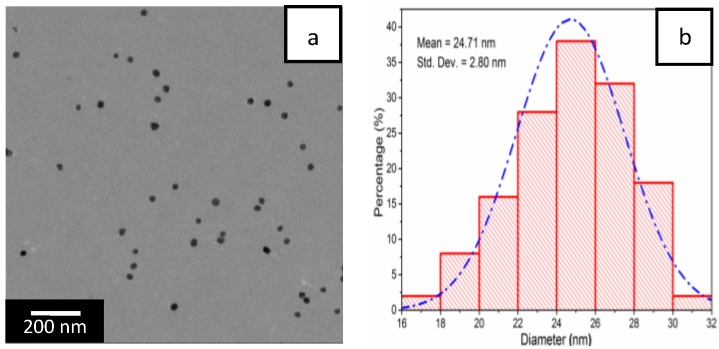

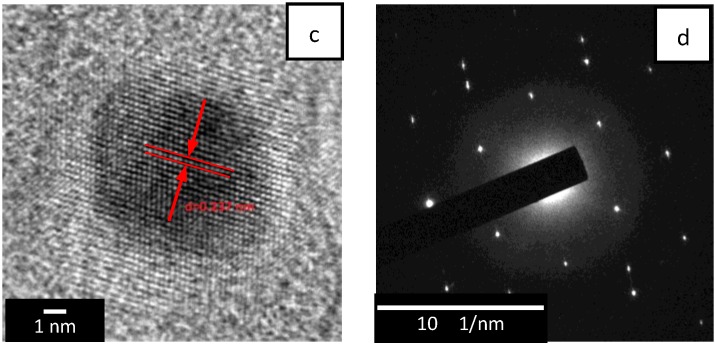
(**a**) Transmission electron microscopy (TEM) image of QCMC-Ag; (**b**) size distribution histogram of Ag NPs; (**c**) inverse fast Fourier transform (IFFT) image and (**d**) selected area electron diffraction (SAED) pattern.

An inspection of the TEM image in Figure 8a shows spherical Ag NPs with small size and uniform shape were dispersed evenly in the QCMC matrix. The size distribution of the Ag NPs was centered in the range of 17–31 nm and the mean diameter was 24.71 ± 2.80 nm, which further confirmed the findings of the UV-Vis results. The inverse fast Fourier transform (IFFT) image of a single particle (Figure 8c) and corresponding selected area electron diffraction (SAED) pattern (Figure 8d) confirmed the crystalline nature of the Ag NPs. In particular, the IFFT image in Figure 8c showed lattice fringe spacing that was determined to be 0.237 nm. This was indexed to the d-spacing of the crystalline silver (111) plane (JCPDS 04-0783). The SAED pattern in Figure 8d showed periodic diffraction spots, indicating that the Ag NPs were monocrystalline.

**Figure 9:**
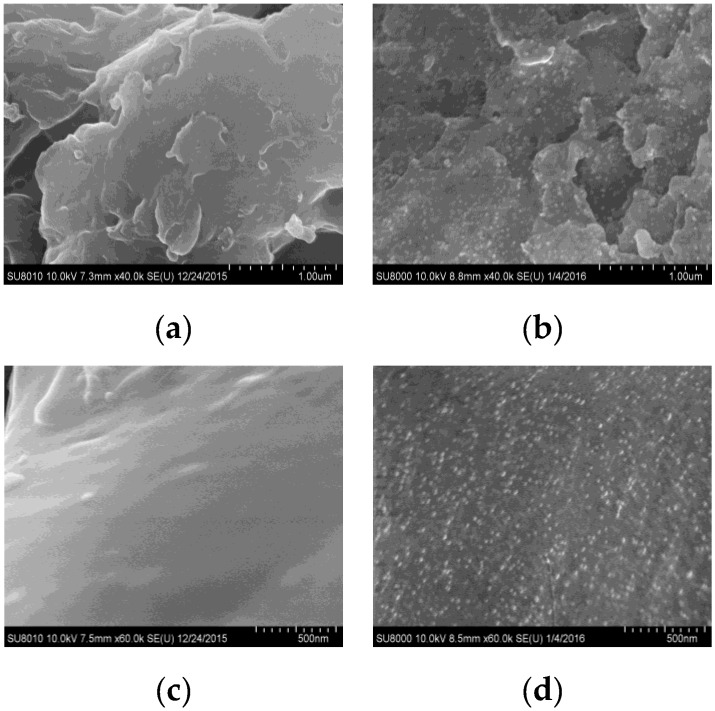
Field emission scanning electron microscope (FESEM) micrographs of (**a**,**c**) QCMC and (**b**,**d**) QCMC-Ag.

The surface morphology of QCMC-Ag NPs composite was further examined using FESEM. Figure 9 displays the FESEM micrographs of the QCMC and QCMC-Ag, in which thousands of silver particles can be seen distributed homogeneously on the surface of the QCMC (Figure 9b,d). The diameter of the particles was similar and almost no agglomeration was observed, which directly demonstrated that QCMC is also an ideal growth template for Ag NPs. The QCMC could reduce Ag^+^ to Ag^0^ due to the functional hydroxyl, carboxymethyl and quaternary ammonium groups in the polymer. During the redox reaction, some of these active groups in the QCMC were oxidized to new groups, but whole polymer chains remained. The remaining QCMC and the oxidized polymer chains acted as an excellent stabilizing agent adsorbing that wrapped the formed Ag NPs tightly to avoid aggregation. Thus, it is not surprising that the Ag NPs were well-dispersed in the QCMC matrix and showed a narrow size distribiution, in agreement with the UV-Vis results and TEM images.

### 3.3. Thermal Stability Analysis

**Figure 10:**
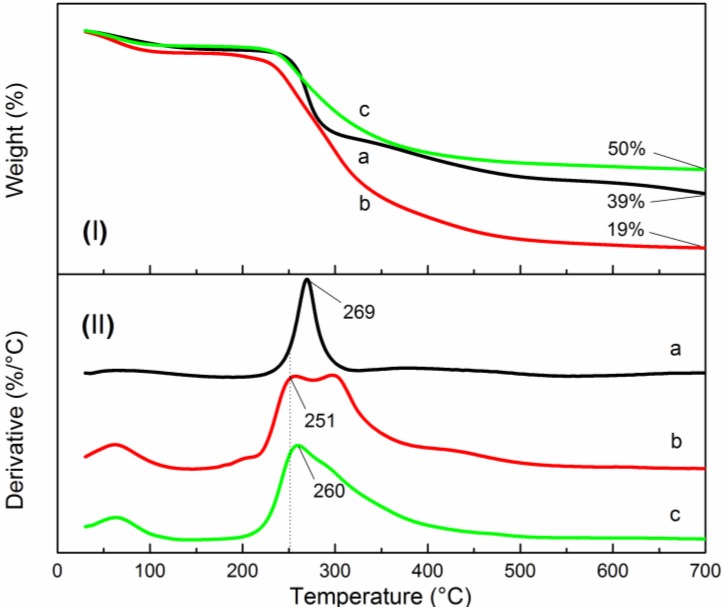
Thermogravimetric (TG) curves of (**a**) CMC, (**b**) QCMC and (**c**) QCMC-Ag, (**I**): thermogravimetric analysis (TGA) curves; (**II**): derivative thermogravimetry (DTG) curves.

The TGA and DTG curves of CMC, QCMC and QCMC-Ag are shown in Figure 10. The thermal decomposition of the two chitosan derivatives and QCMC-Ag were similar, consisting of two stages. The first stage occurred below 100 °C, and is associated with the loss of adsorded and bound water. The second stage (200–400 °C) corresponds to the degradation of the molecular chains of the chitosan derivatives [39]. These results suggest that the entire polymer chain was present in the QCMC-Ag so that the Ag NPs remained stable without aggregating, which is in agreement with the other experimental results reported here.

The TGA curves in Figure 10I show that there was about 4.7% weight loss, corresponding to water loss that occurred below 100 °C for CMC, and an 8.2% loss for the QCMC. This indicates that the QCMC had higher water retention capacity than the CMC due to the presence of the quaternary ammonium groups. Additionally it is noteworthy that the QCMC was more thermally unstable than the neat CMC; only 19% of the QCMC remained up to 700 °C. By contrast, the CMC showed high thermal stability, with a residual weight at 700 °C of 39% and *T*_max_ (the temperature at which the rate of weight loss reaches a maximum) at 269 °C. These results demonstrate that the quaternary ammonium groups grafted on the CMC chains significantly decreased the thermal stability. The *T*_max_ of the QCMC-Ag increased by 9 °C compared to the QCMC and 50% of the QCMC-Ag mass remained at 700 °C, indicating the QCMC-Ag exhibited better thermal stability than the QCMC due to the high thermal stability of Ag NPs.

### 3.4. Antibacterial Activity

**Figure 11:**
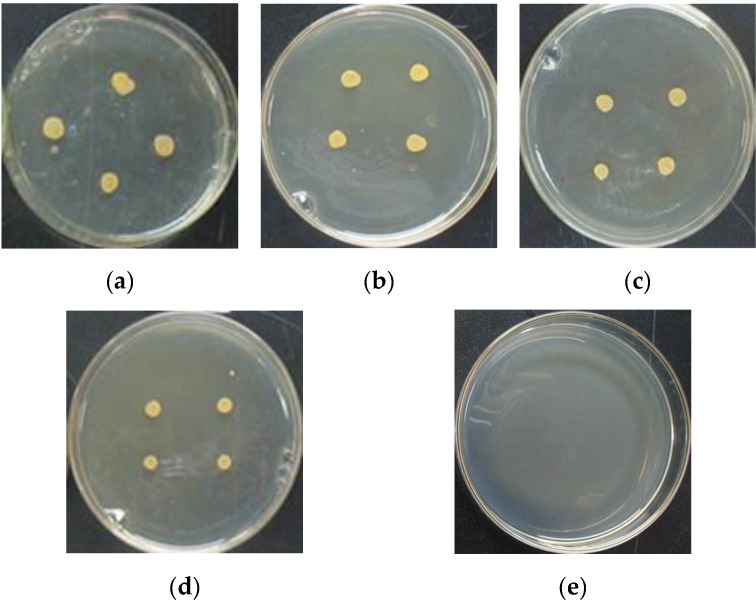
Appearance of colonies of Staphylococcus aureus after treatment with CMC, QCMC , Ag NPs and QCMC-Ag at a concentration of 0.005%: (**a**) blank; (**b**) CMC; (**c**) QCMC; (**d**) Ag NPs and (**e**) QCMC-Ag.

The inhibitory effects of CMC, QCMC, Ag NPs and QCMC-Ag against *Staphylococcus aureus* were measured based on the MIC determinations. As shown in Table 1, pure CMC has a relatively weak inhibition for bacteria, the untreated QCMC showed a slight enhanced inhibitory effect, and pure Ag NPs displayed a relatively strong antibacterial property. Notably, the QCMC-Ag exhibited superior antimicrobial activity. Figure 11 displays the morphology of *Staphylococcus aureus* colonies after treatment with CMC, QCMC, Ag NPs and QCMC-Ag at a concentration of 0.005%, compared to the control. As shown in Figure 11, the QCMC-Ag completely inhibited *Staphylococcus aureus* growth. By contrast, at the same concentration of 0.005%, CMC, QCMC and Ag NPs did not affect the growth of *Staphylococcus aureus*.

The mechanism of the antibacterial activity of the Ag NPs is still a matter of dispute. According to Dallas *et al.*, the three most popular mechanisms proposed are: (a) Ag NPs directly damaging cell membranes, (b) gradual release of free silver ions, followed by disruption of adenosine triphosphate (ATP) production and deoxyribonucleic acid (DNA) replication, and (c) Ag NPs and silver ions generating reactive oxygen species [40]. Lok *et al.* showed that the oxidation of metallic silver to active silver ions species is required for Ag NPs to be fully efficient, since partially oxidized Ag NPs exhibit antibacterial properties and zero-valent nanoparticles do not [41]. Kumar reported that the oxidation of metallic silver to silver ions can be achieved through the interaction of the silver with water molecules [42]. Additionally, electron microscopic analyses showed that Ag NPs can attach to the surface of the cell membrane and thus could damage membrane function or penetrate the bacteria via diffusion or endocytosis [43].

Embedding Ag NPs in a QCMC polymer network enables the sustained release of silver ions and/or silver nanoparticles to exert long-acting and high-efficiency antibacterial activity. The ability of the QCMC to absorb and hold water can facilitate oxidation of Ag NPs to active silver ions. In addition, QCMC is a positively-charged polyelectrolyte and thus can interact vigorously with negatively-charged bacteria at the cell surface, change bacterial cell membrane permeability, restrain the growth of cells, and even kill cells [44]. Since QCMC-Ag can combine both advantages of QCMC and Ag NPs and exhibit a synergistic antibacterial effect, its antimicrobial properties in this study were improved compared to pure QCMC and Ag NPs.

ICP-OES testing showed that the concentration of the silver released from QCMC-Ag in the test solution was extremely low, in the order of 0.12 µg/mL (Table 1), in comparison with pure Ag NPs (1.3 µg/mL). The fact that only small amounts of silver led to antimicrobial activity is a great advantage, because silver ions and/or Ag NPs may exhibit cytotoxic and genotoxic effects on human cells at high concentrations [45].

## 4. Conclusions 

In this study, Ag NPs were synthesized in a QCMC aqueous solution without an additional reducing and stabilizing agent, using silver nitrate as the silver source. The synthesis was conducted in an aqueous medium in the presence of microwave irradiation for 8 min. The results showed that uniform and stable spherical Ag NPs were successfully obtained via a facile, green method. The prepared Ag NPs were well dispersed in a QCMC matrix with a narrow size distribution and a mean diameter of 24.71 ± 2.80 nm. Due to the presence of the Ag NPs, the thermal stability and antibacterial properties of the QCMC-Ag were significantly improved compared to the QCMC. The QCMC-Ag exhibited outstanding hybrid performance with favorable thermal stability, strong antibacterial activity and low human toxicity, which implies that this material might be a promising antimicrobial candidate for application in the biomedical field, food packaging, coating, clothing, textile and other similar areas.

## Figures and Tables

**Table 1:** Minimum inhibition concentrations (MICs) of CMC, QCMC, Ag NPs and QCMC-Ag; the concentration of silver released from Ag NPs and QCMC-Ag.

Sample Code	MICs (%, *w*/*v*, *n* = 3)	Ag Release after 24 h (µg/mL)
Blank	-^1^	-
PBS	-^1^	-
CMC	2	-
QCMC	0.5	-
Ag NPs	0.025	1.3
QCMC-Ag	0.005	0.12

^1^ No antibacterial properties.
